# Data Spine: A Federated Interoperability Enabler for Heterogeneous IoT Platform Ecosystems

**DOI:** 10.3390/s21124010

**Published:** 2021-06-10

**Authors:** Rohit A. Deshmukh, Dileepa Jayakody, Alexander Schneider, Violeta Damjanovic-Behrendt

**Affiliations:** 1 Fraunhofer Institute for Applied Information Technology FIT, 53754 Sankt Augustin, Germany; alexander.schneider@fit.fraunhofer.de; 2 Salzburg Research Forschungsgesellschaft, 5020 Salzburg, Austria; dileepa.jayakody@salzburgresearch.at (D.J.); violeta.damjanovic@salzburgresearch.at (V.D.-B.)

**Keywords:** IoT platform interoperability, Service-Oriented Architecture, Smart Manufacturing, smart factory, industry 4.0, Cloud Manufacturing, IoT ecosystem, federated IoT platforms, digital manufacturing platforms, lot-size-one manufacturing

## Abstract

Today, the Internet of Things (IoT) is pervasive and characterized by the rapid growth of IoT platforms across different application domains, enabling a variety of business models and revenue streams. This opens new opportunities for companies to extend their collaborative networks and develop innovative cross-platform and cross-domain applications. However, the heterogeneity of today’s platforms is a major roadblock for mass creation of IoT platform ecosystems, pointing at the current absence of technology enablers for an easy and innovative composition of tools/services from the existing platforms. In this paper, we present the Data Spine, a federated platform enabler that bridges IoT interoperability gaps and enables the creation of an ecosystem of heterogeneous IoT platforms in the manufacturing domain. The Data Spine allows the ecosystem to be extensible to meet the need for incorporating new tools/services and platforms. We present a reference implementation of the Data Spine and a quantitative evaluation to demonstrate adequate performance of the system. The evaluation suggests that the Data Spine provides a multitude of advantages (single sign-on, provision of a low-code development environment to support interoperability and an easy and intuitive creation of cross-platform applications, etc.) over the traditional approach of users joining multiple platforms separately.

## 1. Introduction

The rapid growth and pervasiveness of the Internet of Things (IoT) is giving rise to a large number of IoT platforms across different application domains. In the manufacturing sector, the industry trends are changing as a result of the increased digitalization and new possibilities are arising for companies to collaborate, exchange data, share services, reuse existing solutions and create innovative products. Many companies are already under constant pressure to react more quickly to changing market demands to create highly customized and interconnected products, facing the need to “federate” their products and tools/services by offering them through existing digital platforms, or by joining platform ecosystems.

The creation of an ecosystem of federated IoT platforms in the manufacturing domain helps companies form agile, ad hoc collaborative networks, establish dynamic supply chains, and optimize production processes to meet new market demands of both Industry 4.0 and lot-size-one manufacturing. The motivation of smart factory companies is to widen their market potential, establish the necessary scale and make their businesses viable.

**Figure 1:**
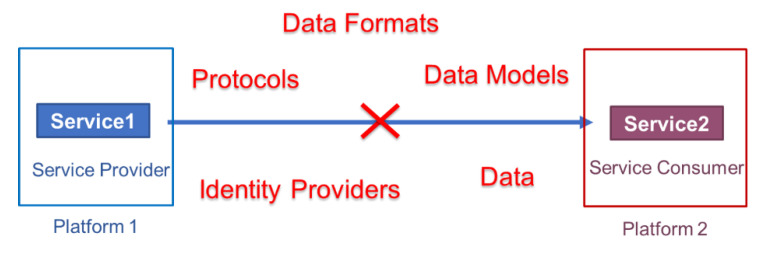
An illustration of interoperability gaps between services of heterogeneous Internet of Things (IoT) platforms.

However, today’s IoT platforms are largely heterogeneous, vendor-specific, vertically oriented, fragmented functionality-wise, and locked behind their own closed identity and access management mechanisms [1,2]. Because of the interoperability gaps between services of different platforms at the levels of interfaces, communication protocols, data formats, data models, identity providers, etc., it is not possible to form composite applications with services from multiple platforms (Figure 1).

This vertically oriented, isolated, and closed nature of platforms leads to inefficient use of resources such as data and services. These platforms are often either domain-centric or vendor-specific and provide limited functionality focused on certain use cases. To take advantage of a wider range of functionality, the consumers must join multiple IoT platforms, resulting in increased costs for them and interoperability issues. As reusability is limited, the cost of joining a platform increases, barring the entry of SMEs and start-ups. This gives an unfair advantage to big companies. In addition, network effects can cause concentration of tools, services, and users around a specific multi-sided IoT platform resulting in monopoly power in IoT market [3]. The coopetition mechanisms such as ‘service provider multihoming’ and ‘consumer switching’ that can prevent high market concentration and monopoly by incentivising competition, become challenging and expensive to practice because of the interoperability issues among IoT platforms [3]. Finally, the lack of interoperability in today’s IoT platforms additionally hinders innovation. Therefore, establishing an ecosystem of such IoT platforms to enable cross-platform, cross-domain communication and collaboration is a challenge that needs to be addressed to cater to service providers’ and consumers’ expectations.

In this paper, we present the Data Spine, a federated platform enabler as a mechanism that interconnects and establishes interoperability between digital manufacturing platforms and enables the creation of a smart factory ecosystem called EFPF (European Factory Platform). The EFPF ecosystem is being established as a part of a European research project called ‘EFPF: European Connected Factory Platform for Agile Manufacturing’ [4]. The main objective of the EFPF project is to create an ecosystem of heterogenous smart factory platforms that enables companies to create and operate ad-hoc collaborative networks in order to meet the market demands for mass-customization. Thus, the EFPF ecosystem is aimed at enabling companies to make a transition from traditional mass production to a lot-size-one manufacturing.

The EFPF ecosystem is initially aimed at interlinking four digital manufacturing platforms from the European Factories-of-Future (FoF-11-2016) cluster focused on supply chains and logistics [5]—namely NIMBLE [6], COMPOSITION [7], DIGICOR [8], and vf-OS [9]. Apart from interlinking the four FoF-11-2016 platforms, which we refer to in this paper as “base platforms”, the EFPF ecosystem is envisioned to be an extendable system capable of interlinking more third-party platforms in the future. Therefore, in order to meet the need for incorporating new tools, services and platforms in the digital platform ecosystem, this paper presents the architecture of the EFPF Data Spine, which is designed at its core to deliver an interoperable, modular and extensible platform solution.

The main contributions of this paper are as follows:

- The paper highlights the need for and the benefits of creating an ecosystem of heterogeneous IoT platforms, identifies the gaps in the state of the art, and presents a new approach that makes use of a federated platform enabler, the Data Spine, to enable the creation of the ecosystem.
- The paper identifies the interoperability and federation requirements for the Data Spine.
- The paper presents the design and a reference implementation of the Data Spine that enables the creation of an ecosystem where:
  ○ The users can seamlessly access tools and services from different platforms using a single set of credentials.
  ○ The users can create innovative cross-platform applications easily and intuitively, with minimal coding effort.
  ○ New tools, services and platforms can be easily integrated with the ecosystem. No local deployments are necessary for their integration or for the creation of composite applications using the existing services.
  ○ The ecosystem administrator does not have to bear the burden of maintaining a common, canonical data model or a common application programming interface (API), but the burden of data transformation is distributed among the service consumers.
  ○ The users from the same or different companies can not only collaborate for developing new applications, but also limit access to their resources, where required.
- Finally, the paper presents two commonly occurring use cases for synchronous request-response and asynchronous Publish/Subscribe (Pub/Sub) communication paradigms in the manufacturing domain and an evaluation of the Data Spine approach which suggests that it provides all the above-listed advantages over the traditional approach of users joining multiple platforms separately to avail the benefits, at the cost of a reasonable performance overhead.

The rest of the paper is organized as follows: in Section 2, we explore other approaches that address the issue of interoperability among heterogeneous IoT platforms and identify the research gap in the state of the art that motivates the design and development of the Data Spine. In Section 3, we identify the interoperability and federation requirements for the Data Spine. In Section 4 and Section 5, respectively, we present the architecture and a reference implementation of the Data Spine. In Section 6, we describe the process of integration of services through the Data Spine. Section 7 presents examples of dataflow through the Data Spine. In Section 8, we provide a performance evaluation of the Data Spine approach. Finally, we discuss the architectural considerations, implications, and the interoperability approach followed by the Data Spine in Section 9 and conclude in Section 10.

## 2. Related Work

In the last few years, the lack of interoperability among IoT platforms has been recognized as one of the major barriers that is preventing the rise of vibrant IoT ecosystems [2,10,11]. In many ways, addressing the problem of IoT platform interoperability is similar to addressing the problem of enterprise integration and interoperability, where different IoT platforms can be seen as enterprises sharing their data, tools, and services. The CEN/ISO 11354 Framework for Enterprise Interoperability [12,13] defines three approaches that can be employed to enable interoperability among the services of heterogeneous platforms, provided by different enterprises:

- Integrated approach: A single data model at the ecosystem level is defined and all connected platforms need to align their tools and services to conform to this common data model.
- Unified approach: A single, non-executable data model is defined at the metadata level for the ecosystem and all connected platforms need to adhere to this meta-model to allow mapping between their models for enabling cross-platform communication.
- Federated approach: There is no common data model or metadata model imposed at the ecosystem level and therefore, the platforms are free to choose any standard or proprietary data model. Connections between tools and services of different platforms are established when required by a use case. Therefore, the partners need to share an ontology to map between their data models, improve interoperability and data sharing.

Many solutions that have been proposed in recent years to address the problem of IoT platform interoperability broadly follow one of these approaches, or a combination of the above approaches. For example, meSchup platform [14] is an example of integrated approach, as it establishes a common, shared API that all connected parties need to conform to. If such an approach is followed to establish an ecosystem of heterogeneous platforms owned by different companies, it would need significant adjustments to the platforms to be connected.

The projects from the European ‘IoT European Platform Initiative (IoT-EPI)’ cluster [15] such as symbIoTe, BIG IoT and INTER-IoT follow either the unified approach, or the federated approach, or a combination of these approaches. The symbIoTe project [10,16] uses a minimal common ontology to enable discovery, whereas the INTER-IoT project [17] uses a central ontology which is maintained in a modularized fashion, and the connected platforms are required to align their metadata models with it.

The symbIoTe project [10,16] provides an interoperability framework to allow cooperation among IoT platforms to establish federations, aiming at the creation of innovative cross-domain applications. It offers mediation services and makes use of systematic development methodologies [18] to address the problem of interoperability across different IoT platforms. However, only the components enabling the registration and discovery of services are located in the cloud and each platform needs to deploy, configure and manage an adapter to connect to other platforms and perform data mapping and transformation.

The BIG IoT project [2,19] is aimed at bridging the interoperability gaps between IoT platforms to enable an ecosystem. It offers a BIG IoT Marketplace for service registration, discovery, accounting, service composition, etc., and client libraries that enable platforms to connect to the BIG IoT API. It uses a data format based on Web of Things (WoT) and WoT Thing Description model [20] to capture the metadata of resources. Similar to the symbIoTe approach, data transformation in BIG IoT is done on the service provider/consumer side. By contrast, the Data Spine is envisioned to be a cloud-native solution that uses API specification standards to capture the metadata of services. Furthermore, the Data Spine aims to provide data transformation tools to facilitate the data transformation process in the cloud.

Moreover, as per the two different authentication modes supported by BIG IoT, either the BIG IoT Marketplace acts as the centralized identity provider granting access to resources or the consumers need to obtain an access token directly from the provider’s platform. In contrast, the Data Spine is envisioned to federate the identity providers of connected platforms and enable single sign-on (SSO) functionality across the ecosystem, offering a distributed security solution in the EFPF ecosystem.

The INTER-IoT project [11,17] aims to enable interoperability between IoT platforms at different IoT system layers. It defines a common API and specifies a methodology for IoT system integration. Like symbIoTe, it addresses the data mapping aspect. However, data mapping needs to be undertaken between connected platforms and a common shared ontology at the ecosystem level. In contrast, the Data Spine does not aim to define a single shared data model. The platforms would be free to choose to map their data models either to any standard data model, depending upon the domain and the use case, or directly to another platform’s data model they want to communicate with. Moreover, INTER-IoT does not explicitly address the issue of federation of existing identity providers of the heterogeneous platforms to form an ecosystem.

Furthermore, there has been substantial research on IoT connectors and gateways in the last few years [21,22]. The IoT gateways that support lower layer protocols and other IoT networking technologies (ZigBee, ZWave, Bluetooth Low Energy (BLE), Long Range Wide Area Network (LoRaWAN), etc.) would be complimentary to the Data Spine as it aims to provide support for standard application layer communication protocols.

In the manufacturing domain, Zeid, et al. [23] describe how the proliferation of technology is causing the evolution of traditionally hierarchical and closed manufacturing architectures towards integrated networks of devices, services, cloud platforms and enterprises. The authors highlight the need for interoperability as one of the major challenges resulting from this evolution. Similarly, Hyoung Seok Kang, et al. [24] conclude that interoperability is one of the most important issues that needs to be addressed in smart manufacturing. Many research projects in the area of smart manufacturing and cloud manufacturing are focused on making data, systems, resources, and other manufacturing infrastructure available as services, without considering aspects towards integration and interoperability with systems from other factories or platforms. For example, Wang et al. [25] propose a framework to provide infrastructure (e.g., robots) as a service, and Chen et al. [26] propose a platform that offers enterprise resource planning (ERP) as a service. However, they do not address how collaboration with other factories or platforms can be enabled.

The solution proposed by Delaram et al. [27] addresses the issue of interoperability among cloud manufacturing enterprises by using integrated data formats. The solution involves decomposing existing services provided by the platforms of different enterprises into generic services, which is followed by mapping these generic services to the basic elements of Electronic Data Interchange (EDI) X12 standards, thereby achieving an abstract common interface. However, the solution presented in [27] neither specifies how the service decomposition and mapping can be achieved and the complexity involved, nor does it address the issue of the mapping of services that are beyond the scope of the EDI X12 standards.

A. Kusiak in [28] considers ‘resource sharing and networking’ as one of the six pillars of smart manufacturing. The author asserts that the decoupling of physical and cyber spaces enables collaboration across business enterprises and predicts that the degree of horizontal connectivity across manufacturing enterprises and inter- and intra-enterprise interoperability will increase.

Tao et al. in [29] make a strong case for the use of services in manufacturing in order to bridge gaps between physical and cyber worlds, enabling platform independence and interoperability, large-scale collaboration among manufacturing enterprises, empowering companies to quickly respond to complex market demands of mass personalization and thus, increasing profit. The authors note that the emergence of new information technology (IT) generation (big data, IoT, cloud computing, etc.) has enabled the manufacturing enterprises to make a rapid shift towards services. These enterprises have now started embracing the concept of manufacturing-as-a-service by offering manufacturing resources as cloud-based services. The Data Spine aims to capitalise on the trend of “servitization” by focusing on enabling interoperability on service-level across digital manufacturing platforms.

## 3. Integration, Interoperability and Federation Requirements for the Data Spine

The EFPF ecosystem is built using a federation approach in mind—the distributed heterogeneous digital platforms managed by different independent entities permit the creation of added value within the ecosystem. To enable communication in the platform ecosystem, a communication layer that acts as a translator/adapter between the heterogeneous tools and services of these platforms needs to be implemented. The communication layer needs to provide API adaptation functionalities, data transformation and routing capabilities, common access methods, and should enable communication without making any modifications to the existing services.

To enable communication and interoperation in the EFPF ecosystem, the initial integration, interoperability, and federation requirements for the Data Spine are compiled as follows:

- **Interoperability**: The Data Spine should support interoperability at the levels of protocols, data models and security.
  ○ **Protocol Interoperability**: The Data Spine should support standard application layer communication protocols from the synchronous (request-response) and asynchronous (Pub/Sub) communication paradigms.
  ○ **Data Model Interoperability**: The Data Spine should bridge the interoperability gaps between services at the levels of data formats, data structures and data models.
  ○ **Security Interoperability**: The Data Spine should facilitate federated security and SSO capabilities in the EFPF ecosystem. It should be possible to call the services of different platforms with a single set of credentials.
- **Federation approach**: The ecosystem should follow a federation approach, enabling “on-demand” interoperability between different tools/services, i.e., when required by a use case. No common data model or format should be imposed, so that there is no overhead on the system administrators of maintaining such a complex canonical model and on the services to understand it and adhere to it.
- **Agility and flexibility**: The ecosystem should allow tools/services to use neutral APIs, which are not strongly tied to any specific implementation. This will allow them to upgrade their APIs without any dependency concerns, thereby giving them the flexibility to evolve independently. The Data Spine should provide an intelligent and flexible infrastructure to align APIs “on-demand”, by creating workflows or “integration flows”.
- **Usability and multitenancy**: The Data Spine should provide an intuitive, low-code development environment to align the APIs of services and enable communication among them. It should be possible for the system integrator users to collaborate, but at the same time to limit access to their integration flows, when required.
- **Built-in functionality and tool/service integration effort**: The Data Spine should take care of the boilerplate code for protocol translation, routing, and mediation, etc., and facilitate the system integrator users for integrating their services by configuring only the service-specific parts of their integration flows with minimal coding effort.
- **Platform integration effort**: No local deployments of any Data Spine components should be needed to integrate 3rd party platforms in the ecosystem.
- **API management**: The system integrator users need to refer to the technical specifications of service APIs to create integration flows. The Data Spine should provide a component to store and retrieve service metadata including the API specifications. That component should ensure uniformity across and completeness of the API specifications.
- **Modularity and extensibility**: The architecture of the Data Spine should be designed with modularity and extensibility in mind to meet the need for incorporating new tools, services, and platforms in the EFPF ecosystem, with minimum effort.
- **Performance, scalability, and availability**: As the Data Spine is a central entity of the platform ecosystem, it should be highly performant and should support high throughput. The performance critical components of the Data Spine should have the capability to operate within a cluster to support high availability.
- **Maintainability**: In the view of maintainability, the Data Spine should facilitate the creation of a loosely coupled, modular and an easily extensible ecosystem.

**Figure 2:**
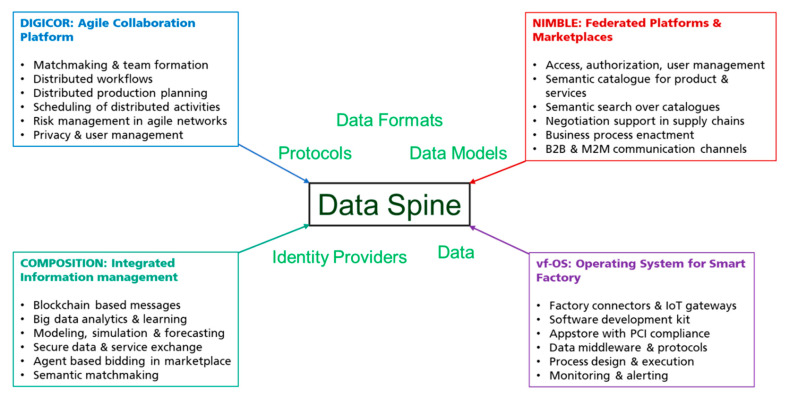
Conceptual overview of the EFPF (European Factory Platform) ecosystem.

Along with the initial guiding requirements for establishing a federated platform ecosystem listed above, the concrete interoperability requirements for the design and realisation of the Data Spine are derived from the four base platforms in the EFPF project. These four platforms functionally complement each other, offering services with minimum overlap (Figure 2).

The technical profiles of the four base platforms are documented, which included the specification of their tools, services and components, their maturity levels, exposed interfaces, protocols, data models, data formats, access control mechanisms, authentication providers supported, dependencies, programming environment, technical documentation, etc. A summary of the technical profiles of these platforms is presented in Table 1.

Based on the final collection of requirements for establishing a federated platform ecosystem, the conceptual components of the Data Spine are defined. The subsequent sections describe the design and architectural aspects of these individual conceptual components and their interrelationships.

## 4. Design of the Data Spine

**Figure 3:**
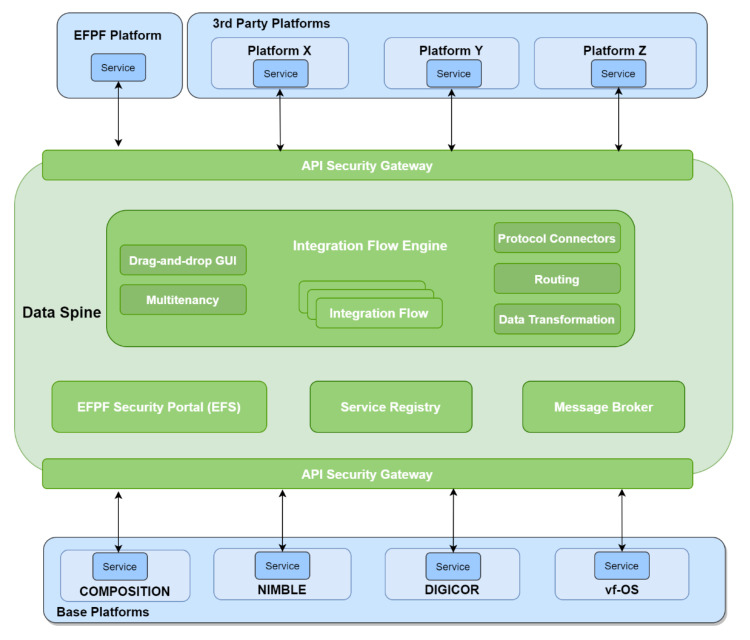
High-level architecture of the Data Spine.

Figure 3 illustrates the high-level architecture of the Data Spine enabling communication across different platforms in the EFPF ecosystem, following the service-oriented architecture (SOA) pattern.

The Data Spine represents a collection of the following conceptual components that work together to form an integration, interoperability, and communications layer for the EFPF ecosystem:

- Integration Flow Engine;
- API Security Gateway;
- Service Registry;
- Message Broker;
- EFPF Security Portal (EFS).

### 4.1. Components of the Data Spine

This section describes the core conceptual components of the Data Spine, their functionality, and the role they play in establishing the EFPF ecosystem and enabling cross-platform communication.

#### 4.1.1. Integration Flow Engine

The integration flow engine (IFE) is the component of the Data Spine that provides service integration and interoperability infrastructure with capabilities such as connectivity, data routing, data transformation and system mediation. These capabilities can be used to bridge the interoperability gaps at both protocol and data model levels between the heterogeneous services that communicate through the Data Spine.

The IFE is a dataflow management system based on the concepts from flow-based programming [30] that makes use of workflows/dataflows to interlink services and enable interoperation. In the context of the EFPF ecosystem, such workflows/dataflows are termed as ‘Integration Flows’. The IFE facilitates the lifecycle management, persistency, and execution of the integration flows.

The integration flows are designed and implemented as directed graphs that have ‘processors’ at their vertices and the edges represent the direction of dataflow. The processors are of different types, depending upon the functionality they provide. For example, the processors of type ‘Protocol Connector’ address the issue of interlinking the services that use heterogeneous communication protocols, the processors of the type ‘Data Transformation Processor’ provide means for transforming between data models and message formats, etc. The edges that represent the flow of information support routing of data based on certain parameters, e.g., path parameters and/or query parameters contained in the Uniform Resource Locator (URL) path, Hypertext Transfer Protocol (HTTP) headers, etc.

An instance of the IFE should have in-built protocol connectors for standard communication protocols, which are widely used in the industry. Furthermore, it should have in-built support for data transformation processors that make use of existing data transformation languages such as extensible stylesheet language transformations (XSLT) [31]. The processors are the extension points of the IFE. For instance, to support a new protocol, a new protocol connector needs to be written and added to the IFE.

In addition, the IFE offers an intuitive, drag-and-drop style Web-based graphical user interface (GUI) which can be used to create the integration flows based on the concepts from visual programming [32] paradigm. The IFE and its GUI support multitenancy. The GUI can be configured based on the defined access control policies to allow or restrict visibility of and/or access to certain GUI elements. In addition, access control policies, such as a user or a user group being able to view and manipulate only the integration flows created by him/her/them, can also be defined, and enforced. Thus, the GUI of the IFE supports multitenancy and enables collaboration among system integrator users who create the integration flows. Moreover, the IFE supports standard authentication protocols such as OpenID Connect (OIDC) to secure access to its GUI using a pluggable authentication provider such as Keycloak [33]. This ensures authentication of the same users from the EFS, the identity provider for EFPF.

Finally, to ensure high availability, throughput and low latency, an instance of the IFE should be scalable and capable of operating in a clustered fashion.

#### 4.1.2. Application Programming Interface (API) Security Gateway

The API security gateway (ASG) acts as the policy enforcement point (PEP) for the Data Spine, enabling secure communication among platforms. The ASG intercepts all the traffic to the Data Spine and invokes the security service ’EFPF Security Portal (EFS)’ for authentication and authorization decisions. The ASG automatically creates reverse-proxy endpoints for the API endpoints of services, which are registered in the Service Registry of the Data Spine. Fine-grained access control policies for these proxy endpoints can then be configured in the EFS.

#### 4.1.3. Service Registry

**Figure 4:**
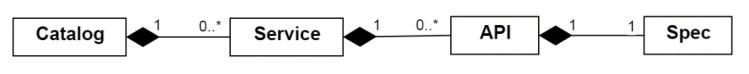
Abstract class diagram for the service registry.

In a federated platform ecosystem, service discovery, integration and orchestration should be possible across different platforms, enabling those platforms to achieve common objectives. The service registry component of the Data Spine enables the service providers in the EFPF ecosystem to register and advertise their services. It also enables the service consumers or system integrator users to discover these services and retrieve their functional and technical metadata, such as the API specifications. Figure 4 illustrates an abstract class diagram for the service registry. The description of an abstract service schema is presented in Table 2.

The abstract class diagram for the service registry (Figure 4) shows composition relationship between its classes. The notation ‘0..*’ in the diagram denotes ‘zero or more instances’ of the concerned entity. As illustrated in the diagram, the ‘Catalog’ of the service registry can have zero or more services, each ‘Service’ has zero or more APIs, and each ‘API’ has exactly one ‘Spec’ (specification). Table 2 further shows the abstract schema for the Service object. The API Spec is obtained from an API Spec document, which needs to conform to one of the following standards in order to ensure uniformity across and completeness of API specifications:

- For synchronous (request-response) services: OpenAPI Specification [34];
- For asynchronous (Pub/Sub) services: AsyncAPI Specification [35].

Such design makes the schema capable of managing metadata for synchronous (request-response) as well as asynchronous (Pub/Sub) type of services. All the technical metadata for the APIs of services that is needed for creating the integration flows, can be obtained from the API Spec documents.

The ‘type’ field can be used to categorise the services by introducing a service type, based on the functionality they offer. Any additional functional metadata related to the services or the individual APIs can be stored in the respective ‘meta’ objects as key-value pairs. Thus, the basic schema can be extended to include additional metadata for the entire service or for a specific API. Moreover, the ‘doc’ field can be used to provide a link to further service metadata documentation.

#### 4.1.4. Message Broker

The message broker enables asynchronous (Pub/Sub) communication in the EFPF ecosystem. The factory connectors/IoT gateways installed in different factories publish shop floor data as messages to the message broker to make the data available to the service consumers or subscribers. The message broker supports multitenancy and fine-grained access control.

The message broker provides interfaces for user and topic administration, management, and monitoring. An instance of the message broker should have an in-built support for standard Pub/Sub-based messaging protocols such as Message Queuing Telemetry Transport (MQTT), Advanced Message Queuing Protocol (AMQP), etc., that are widely used in the industry. In addition, the Message Broker can be extended to support new protocols using a plugin mechanism.

#### 4.1.5. European Factory Platform (EFPF) Security Portal (EFS)

In the EFPF ecosystem, each platform has its own identity and access management solution, with defined and stored users, roles, policies, etc., for that platform. To enable collaboration and data exchange among the platforms, it should be possible for a user/tool/service of one platform to call a service of another platform. An SSO functionality needs to be enabled across all platforms in the EFPF ecosystem.

EFS is defined in [36] as the federated identity and access management layer that bridges the “security interoperability” gaps between platforms in the EFPF ecosystem. It federates the identity providers of all platforms in the EFPF ecosystem in order to enable SSO functionality and enables users to seamlessly access tools and services from different platforms using a single set of credentials.

The EFPF ecosystem is envisioned to be an extensible platform ecosystem. As described in [36], providing a completely distributed, federated solution with login options for all platforms in the Web portal of a single platform is not a scalable solution. Therefore, EFS takes on the role of a central identity and access management solution for the EFPF ecosystem and, the Web portal of each platform provides an additional “Login with EFPF” option to allow logging in with an EFPF user account. Furthermore, the EFS together with the ASG can be used to secure the API endpoints exposed by the integration flows.

Finally, to federate the identity provider of a new platform with the EFS, the roles and access policies defined in the new platform’s identity provider need to be aligned with those of the EFS. The EFPF ecosystem can also be extended by adding new tools/services that do not belong to any platform and, hence, do not have their own identity provider. In such a case, these services are added to the ‘EFPF platform’ and EFS directly acts as their identity and access management solution.

### 4.2. The Data Spine Architecture and Components’ Interaction

**Figure 5:**
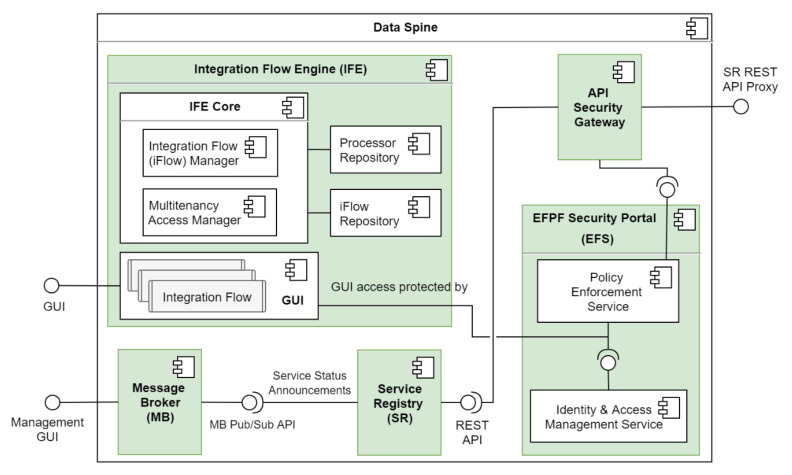
Detailed architecture of the Data Spine.

Figure 5 illustrates the core conceptual components of the Data Spine, including the relationships and interactions between them. Firstly, the access to the GUI of the IFE is protected by the EFS. The ASG relies on the ‘policy enforcement service’ of the EFS to make access control decisions. The ASG is configured to check the service registry for new service registrations and updates to existing services periodically, in order to automatically create secure proxy endpoints for protecting access to their APIs. The access to the Representational state transfer (REST) API of the service registry is secured through the proxy endpoints in the ASG. The service registry publishes service status announcement related messages to the message broker.

The GUI of the IFE loads the built-in processors on start up. The users interconnect the instances of these processors as required to create integration flows. The integration flows are persisted in the iFlow repository of the IFE. The runtime access to the endpoints exposed by the integration flows in the IFE is protected through the corresponding proxy endpoints in the ASG after they are registered in the service registry.

**Figure 6:**
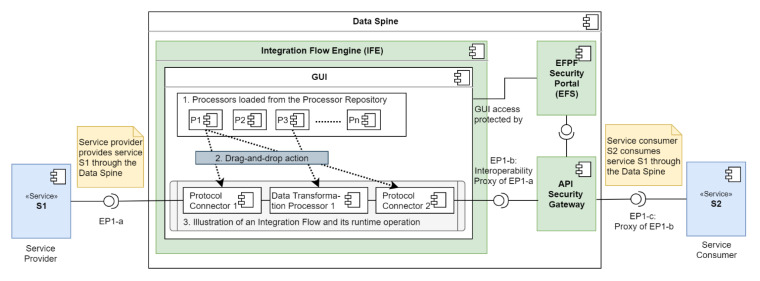
Illustration of the creation and runtime operation of an integration flow.

Figure 6 illustrates an integration flow that bridges the interoperability gaps at the data model level, enabling communication between two services, S1 and S2. The service provider’s service ‘S1′ exposes the API endpoint ‘EP1-a’ and the service consumer’s service ‘S2′ intends to consume it. However, this cannot be done directly due to the different data models that need to be transformed and aligned beforehand. To enable communication, a new integration flow is created and run as follows:

1. Reusable built-in processors: The GUI displays the built-in processors.
2. Creation of the integration flow: The instances of these processors are added to the workspace using ‘drag-and-drop action’ and interconnected as required to create the integration flow.
3. Runtime operation of the integration flow: The integration flow performs the required data model transformation and exposes an “interoperability proxy” endpoint of EP1-a called ‘EP1-b’. A secure proxy of EP1-b called ‘EP1-c’ is automatically created in the ASG, which is then consumed by the service S2.

Thus, the use of visual drag-and-drop style GUI makes the creation of integration flows easy and intuitive. As the built-in processors are reused for protocol connection and data transformation, no source code needs to be written for the creation of integration flows. The ASG automatically creates secure proxy endpoints for the APIs exposed by the integration flows. These functionalities provided by the Data Spine take care of most of the boilerplate code and enable the creation of integration flows with minimal coding effort. In this way, the components of the Data Spine work together to enable the integration of and communication between the services of different platforms.

In summary, the Data Spine provides the following functionalities:

- Authentication, authorization and SSO;
- Service/API metadata lifecycle management and discovery;
- Infrastructure and tooling for protocol connection, data transformation, routing, and system mediation;
- Multitenant, Web-based, drag-and-drop style GUI for an easy and intuitive creation of applications with minimal coding effort;
- Message brokering.

## 5. Reference Implementation of the Data Spine

**Figure 7:**
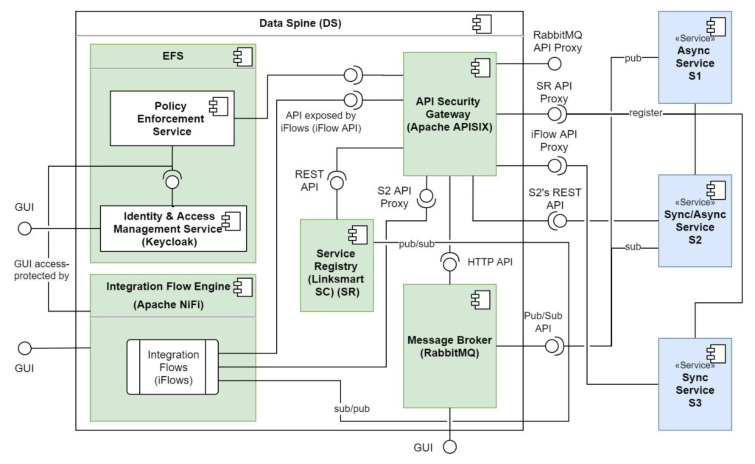
Reference implementation of the Data Spine.

Table 3 lists the technological tools and services used to realise the conceptual components of the Data Spine, their versions used for the deployment and URLs to their source code repositories. Figure 7 further illustrates the relationships and interactions among these components and shows how different synchronous and asynchronous services (S1, S2 and S3) interface with the components of the Data Spine. The subsequent Section 5.1, Section 5.2, Section 5.3, Section 5.4, Section 5.5 introduce these technologies and their role in the EFPF ecosystem.

### 5.1. Integration Flow Engine: Apache NiFi

**Figure 8:**
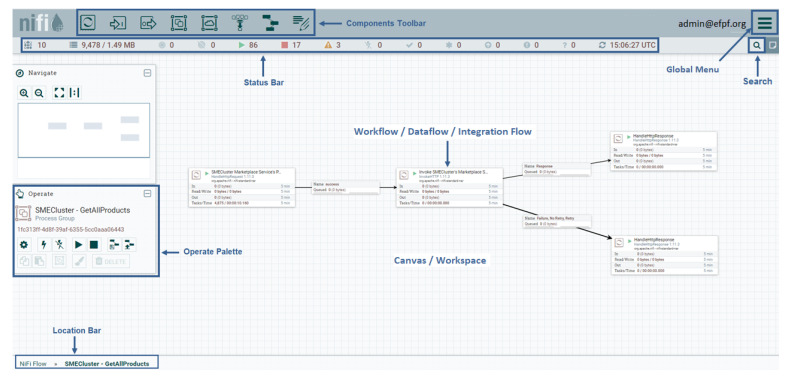
Components of Apache NiFi’s graphical user interface (GUI) and a sample workflow.

The IFE of the EFPF ecosystem is built on top of Apache NiFi [37], a dataflow management platform based on the concepts of flow-based programming [30]. Apache NiFi automates the flow of information between systems through directed graphs called dataflows. The dataflows support communication, data routing, data transformation and system mediation logic with the help of so-called ‘processors’. The processors are responsible for handling data ingress, egress, routing, mediation, and transformation. Apache NiFi offers a Web-based, multi-tenant, highly configurable, drag-and-drop style GUI for creating such dataflows. Figure 8 highlights the components of Apache NiFi’s GUI and shows a sample dataflow. The Data Spine integration flows are realised through the dataflows in Apache NiFi.

Moreover, Apache NiFi provides connectors for standard communication protocols such as HTTP, MQTT, AMQP, etc., which are widely used in the manufacturing sector. In addition, it provides support for data transformation through tools/languages such as Jolt [46], XSLT [31], Java virtual machine (JVM) scripting languages, etc. Its functionality can be extended by developing new custom processors. Apache NiFi also supports scaling-out though the use of clustering to ensure high performance and availability.

### 5.2. API Security Gateway: Apache APISIX

The ASG of the EFPF ecosystem is built on top of Apache APISIX [39], a cloud-native, high performance, dynamic microservices API gateway that supports API traffic management features, such as reverse proxying, load balancing, authentication, dynamic routing, dynamic upstream, hot plugin loading, service governance, etc.

In the EFPF ecosystem, Apache APISIX is deployed with two plugins: (1) Open ID Connect plugin that provides token introspection functionality and (2) Policy Enforcement plugin that enables Apache APISIX to delegate the authorization decisions to the EFS. Moreover, the ASG checks the service registry of EFPF for new service registrations/updates to existing services periodically and creates proxy routes automatically for the registered service APIs.

### 5.3. Service Registry: LinkSmart Service Catalog

The Service Registry is realised using the LinkSmart Service Catalog (SC) [41]. The SC is the entry point for Web services. Its functionality mainly covers the lifecycle management of services i.e., the registration, viewing, updating and deregistration of services’ metadata. In addition, it supports browsing of the service entries in its index page (Catalog) and provides a service filtering functionality that can be used by service consumers to search services by known capabilities. The SC also offers an MQTT API for announcing the service registration/deregistration events over predefined MQTT topics.

The schema of the SC is based on the abstract service registry schema (Table 2) and is capable of managing technical and functional metadata for synchronous (request-response) as well as asynchronous (Pub/Sub) type of services.

### 5.4. Message Broker: RabbitMQ

RabbitMQ [43], a message-oriented middleware that implements AMQP 0-9-1, is used to realise the Message Broker of the Data Spine. RabbitMQ supports AMQP 0-9-1 inherently and MQTT/MQTTS via a plugin. It also supports STOMP (simple (or streaming) text-orientated messaging protocol), AMQP 1.0, HTTP and WebSockets. RabbitMQ provides a management GUI and an HTTP-based API for administration, management and monitoring of channels/topics, users, dataflow stats, etc., via a plugin. RabbitMQ also supports multi-tenant authorization with the help of ‘virtual hosts’ which enable logical grouping and separation of resources such as connections, exchanges, queues, bindings, user permissions, policies, etc. RabbitMQ supports clustered deployment for high availability and throughput. It supports extension of functionality through the use of plugins.

### 5.5. EFS: Keycloak and Other Microservices

The role of EFS in the EFPF ecosystem is to manage users, roles, policies, etc., using Keycloak [33], which is an open-source Identity and Access Management solution. Keycloak makes use of the OpenID Connect protocol [47], which is an authentication layer on the top of OAuth 2.0 authorization protocol [48]. Keycloak of the EFS is added as a trusted identity provider in the identity providers of all the platforms with the aim to enable SSO across the EFPF ecosystem. This further enables creation of linked EFS users in the identity providers of the platforms in the EFS ecosystem [36].

EFS makes use of a microservice called the ‘policy enforcement service’ to enforce the policies defined in Keycloak. The ASG invokes the API of the Policy Enforcement Service for authentication and authorization related decisions.

## 6. Service Integration through the Data Spine

The EFPF ecosystem can be extended in two different ways: (1) by adding new platforms that have their own identity providers and, (2) by adding new tools/services to the EFPF platform which uses the EFS as its identity provider. Integration of a new platform with the EFPF ecosystem needs integration of its identity and access management solution with the EFS as explained in Section 4.1.5. In the latter case of adding a new service to the EFPF platform, a secure proxy API for the service needs to be created and the access control policies for this secure proxy API need to be defined in the EFS. This section focuses on this latter case. It lists the activities that the service providers are required to do in order to provide their services through the Data Spine and the activities the service consumers are required to do in order to consume the services provided through the Data Spine. These design-time aspects become the prerequisites to enabling communication through the Data Spine.

While the Data Spine supports synchronous as well as asynchronous communication paradigms, it is agnostic to the communication patterns employed by the composite applications developed or integrated through it. The decision of which communication pattern to use is made by the providers of tools/services or the developers of composite applications. The design-time service integration activities depend upon the communication mode used. Therefore, the following subsections discuss both ‘synchronous communication’ and ‘asynchronous communication’ in more details.

### 6.1. Synchronous Communication

**Figure 9:**
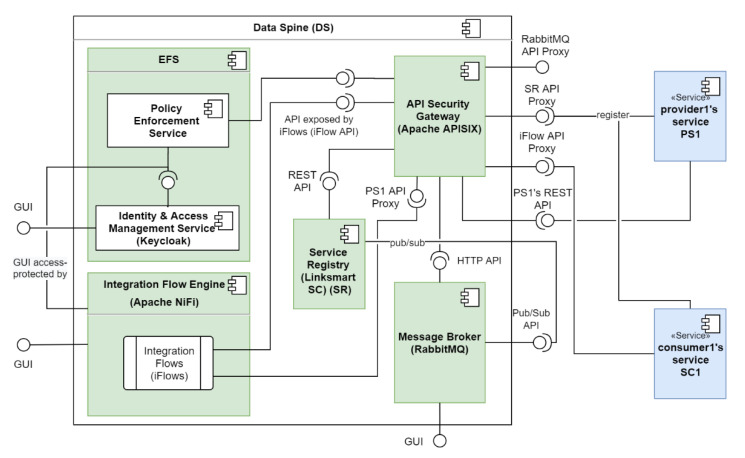
Synchronous services’ integration through the Data Spine.

Figure 9 shows how provider1′s service ‘PS1′ and consumer1′s service ‘CS1′ interact with the components of the Data Spine to provide and consume services, respectively. The actions to be performed for service provision and consumption through the Data Spine are described below.

**Prerequisites:**

- The service provider ‘provider1′ and service consumer ‘consumer1′ are both EFS users.
- provider1 and consumer1 have the necessary permissions required to access the service registry.

**Design-time service integration activities:**

**Figure 10:**
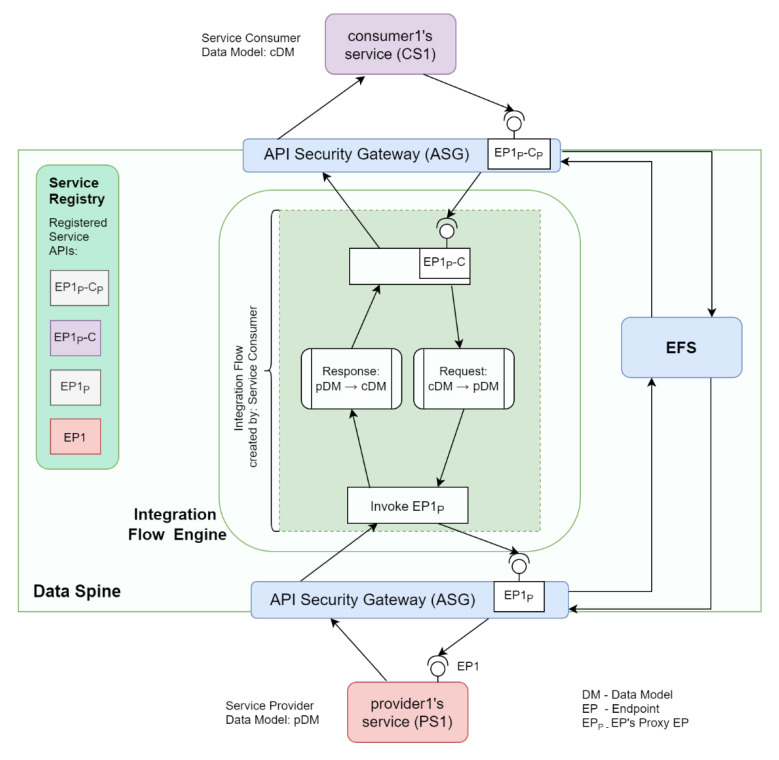
Example of synchronous communication workflow through the Data Spine.

1. Service Registration: provider1 registers his/her service ‘PS1′ to the service registry with an appropriate service ‘type’ (e.g., ‘platform1.marketplace-service’). Let us assume that PS1′s REST API endpoint is ‘EP1′.
2. Creation of a Secure Proxy API: The ASG checks for new service registrations/updates to existing services in the service registry periodically, and automatically creates a secure proxy API endpoint/route ‘EP1_P_’ for EP1 in ASG.
3. Access Configuration: An EFPF administrator user defines/configures the access permissions for accessing EP1_P_ in the EFS.
4. Service Lookup and Metadata Retrieval: consumer1 uses service registry’s filtering API to find PS1, decides to consume it, and retrieves its technical metadata including its API spec from the service registry.
5. Access Configuration: consumer1 requests for and acquires the necessary access permissions to invoke EP1_P_.
6. Access Configuration: consumer1 requests for and acquires the necessary access permissions to create integration flows in the IFE.
7. Integration Flow Creation: consumer1 creates an integration flow using the GUI of the IFE that invokes EP1′s secure proxy endpoint EP1_P_, performs data transformation to align request/response payload to its own data model/format, and finally creates and exposes an “interoperability-proxy” endpoint EP1_P_-C for EP1_P_.
8. Service/API Registration: consumer1 registers this new EP1_P_-C API endpoint to the Service Registry.
9. Creation of a Secure Proxy API: ASG automatically creates a secure proxy API endpoint EP1_P_-C_P_ for EP1_P_-C.
10. Access Configuration: consumer1 requests for and acquires the necessary access permissions to invoke EP1_P_-C_P_.
11. Integration Complete: provider1′s service PS1 and consumer1′s service CS1 are now integrated through the Data Spine and CS1 can start invoking PS1 and obtain a response in the format required by it as illustrated in Figure 10.

### 6.2. Asynchronous Communication

**Figure 11:**
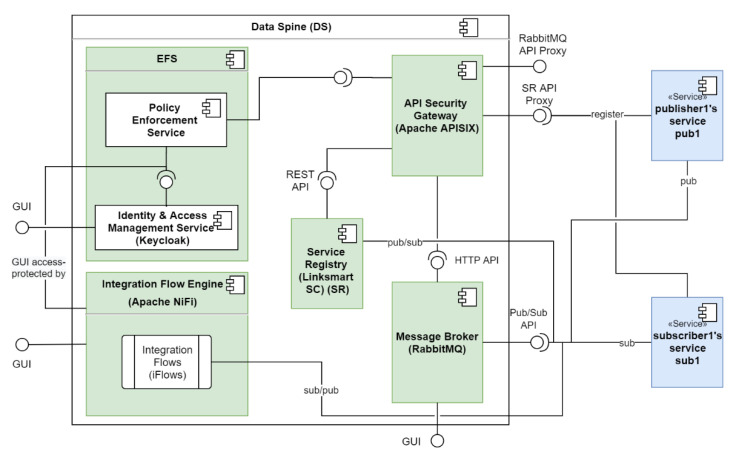
Asynchronous services’ integration through Data Spine.

Figure 11 shows how publisher1′s service ‘pub1′ and subscriber1′s service ‘sub1′ interact with the components of the Data Spine to provide and consume services, respectively. The actions to be performed for service provision and consumption through the Data Spine are described below.

**Prerequisites:**

- The service provider ‘provider1′ and service consumer ‘consumer1′ are both EFS users.
- provider1 and consumer1 have the necessary permissions required to access the service registry.

**Design-time service integration activities:**

**Figure 12:**
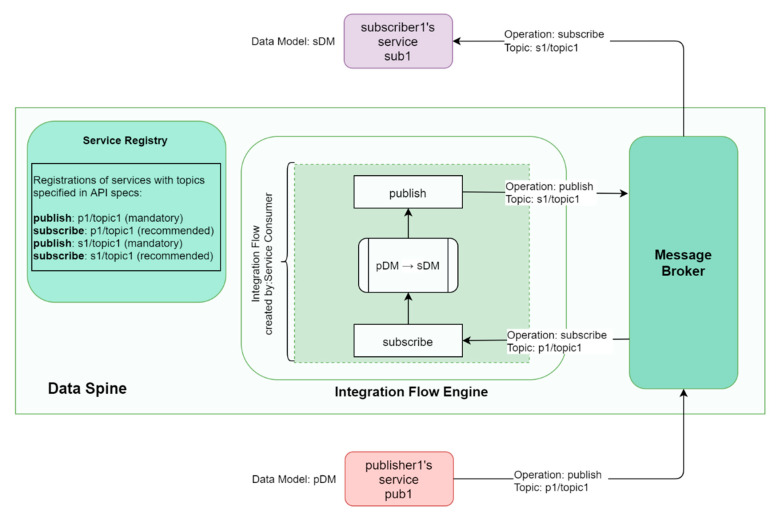
Example of asynchronous communication workflow through the Data Spine.

1. Access Configuration: publisher1 requests for and acquires the necessary access permissions to publish to the message broker over topic ‘p1/topic1′.
2. Publisher Configuration: publisher1 configures his/her service ‘pub1′ to publish to the message broker over the topic ‘p1/topic1′.
3. Service Registration: publisher1 registers pub1 that consists of this Pub/Sub API containing its publication information to the service registry.
4. Service Lookup and Metadata Retrieval: subscriber1 uses service registry’s filtering API to find pub1, decides to subscribe to pub1′s topic ‘p1/topic1′ and retrieves the technical metadata for pub1 including its API spec from the service registry.
5. Access Configuration: subscriber1 requests for and acquires the necessary access permissions to subscribe to p1/topic1 and to publish back to the message broker over a new topic ‘s1/topic1′.
6. Access Configuration: subscriber1 requests for and acquires the necessary access permissions to create integration flows in the IFE.
7. Integration Flow Creation: subscriber1 creates an integration flow using the GUI of the IFE to subscribe to p1/topic1, perform data model transformation to align the message payload to its own data model/format, and finally to publish the resulting data to the message broker over the topic s1/topic1.
8. Service Registration: subscriber1 registers his/her service with the APIs containing its subscription and publication information to the service registry.
9. Integration Complete: publisher1′s service pub1 and consumer1′s service sub1 are now integrated through the Data Spine and, sub1 can subscribe to the topic s1/topic1 and obtain data in the format required by it as illustrated in Figure 12.

## 7. Examples of Dataflow through the Data Spine

This section describes both synchronous and asynchronous dataflow through the Data Spine using two use case examples related to the EFPF ecosystem: integrated marketplace, and anomaly detection and alerting.

### 7.1. Synchronous Dataflow Use Case in the EFPF Ecosystem: Integrated Marketplace

**Figure 13:**
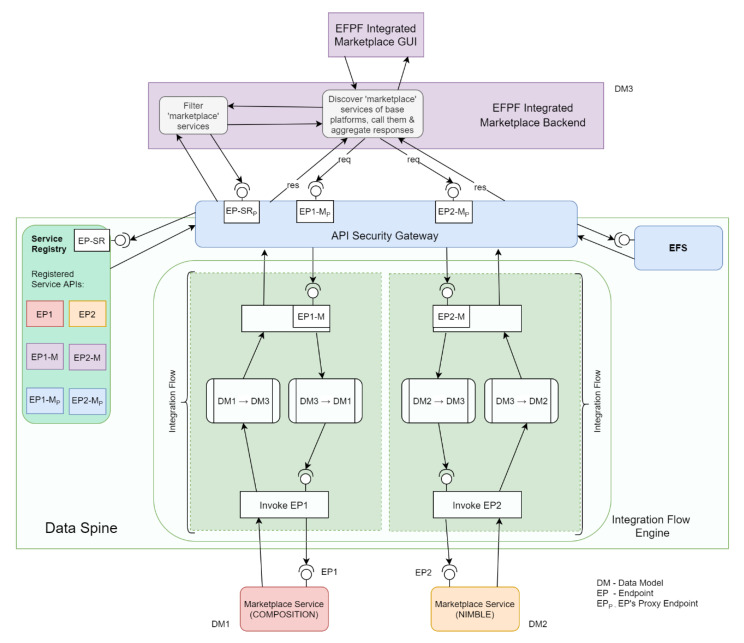
Realisation of an integrated marketplace solution in the EFPF ecosystem.

Figure 13 illustrates the realisation of an integrated marketplace for the EFPF ecosystem that displays an aggregated list of products and services from all the connected platforms. The integrated marketplace obtains this list of products and services from the marketplace services of the connected platforms through the Data Spine and displays them onto the integrated marketplace GUI. In this use case, the data model and security interoperability functionalities of the Data Spine are utilised. For the sake of simplicity, Figure 13 displays only two connected platforms: COMPOSITION and NIMBLE.

To obtain the list of products and services, the integrated marketplace’s GUI initiates a call to its backend. The backend queries the service registry for services of type ‘marketplace’ and retrieves the API endpoints for the marketplace services of COMPOSITION and NIMBLE platforms. It then invokes these services through the integration flows created in the IFE and obtains a response. The data models of responses from these marketplace services are transformed to conform to the integrated marketplace’s data model through the integration flows. Finally, the integrated marketplace’s backend aggregates the responses, and hands over the aggregated list of products and services to the GUI which then displays it.

### 7.2. Asynchronous Dataflow Use Case in the EFPF Ecosystem: Anomaly Detection and Alerting

**Figure 14:**
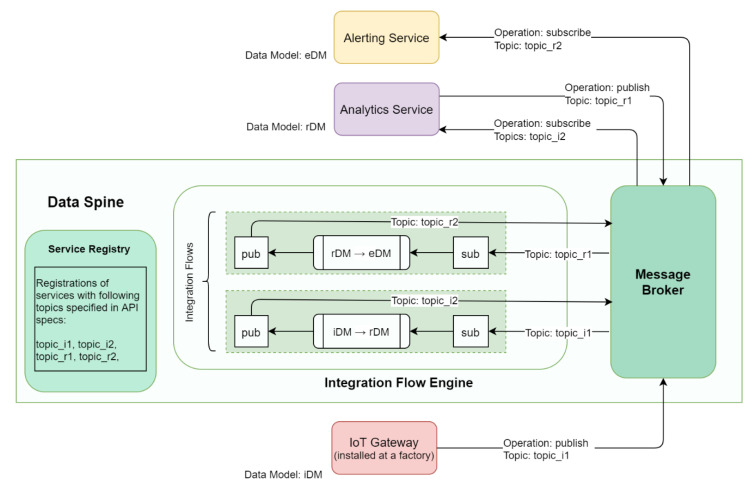
Realisation of a composite anomaly detection and alerting application in the EFPF ecosystem.

Figure 14 illustrates the realisation of a composite application that performs data analysis to detect anomalies and triggers an alert on detection of an anomaly.

In this example, an IoT Gateway installed at a factory collects shop floor data with the help of sensors and publishes it to the message broker of the Data Spine. Through the pre-configured integration flows, this data is transformed to align with the analytics service’s data model, and it is published again to the message broker over a new topic. The analytics service subscribes to this new topic and obtains the data. It then analyses the data to identify possible anomalies, that can further be used for predictive maintenance, fault detection, optimization of a production process, or detection of a safety related risk, etc. Once the analytics service identifies an anomaly, it publishes the data for an alert to be triggered to the message broker. Again, through another pre-configured integration flow, this data is transformed to align with the alerting service’s data model and is published to the message broker over a new topic. The alerting service subscribes to this new topic, gets the data, and triggers an alert. Thus, such a set of tools and services from different platforms can be used to realise a composite application in the EFPF ecosystem through the Data Spine.

## 8. Validation and Results

**Figure 15:**
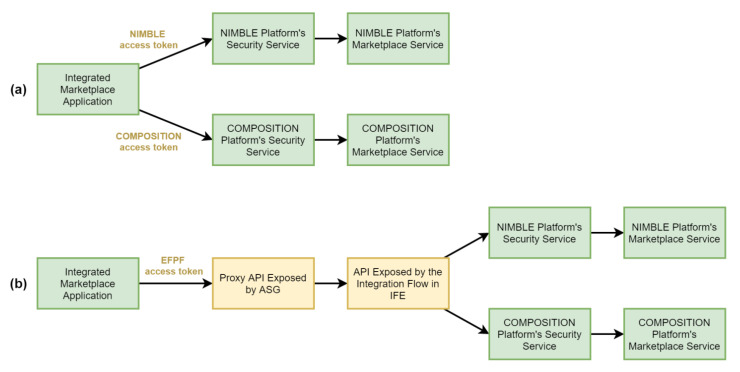
High-level workflows and API calls for realising the integrated marketplace solution: (**a**) the traditional approach, and (**b**) the Data Spine approach.

In this section, we evaluate the performance of the Data Spine approach by using the integrated marketplace use case example described in Section 7.1. In the realisation of the integrated marketplace solution, the Data Spine enables invoking the endpoints of the base platforms’ marketplace services with a single set of EFPF credentials and facilitates the data model transformation process. Without the Data Spine, in the traditional approach, the developer of the integrated marketplace needs to obtain user accounts for each of the base platforms, use those separate sets of credentials for invoking the individual marketplace services of the base platforms and write additional source code to perform the data model transformation locally. If specialised data model transformation tools are used locally, their deployment also needs to be managed separately. The high-level workflows and API calls required for getting a response from the base platforms’ marketplace services in the format expected by the integrated marketplace for (a) the traditional approach, and (b) the Data Spine approach are shown in Figure 15.

We performed a quantitative evaluation of both approaches where the response times were measured as a sum of the time taken for calling NIMBLE platform’s marketplace service and for transforming the response to adhere to the Integrated Marketplace’s data model. The integrated marketplace applications were realised as Java programs that recorded the response times. The Data Spine components were realised using the technologies elaborated in Section 5. Apache NiFi, LinkSmart Service Catalog and RabbitMQ were deployed on a machine with 2 vCPUs and 8 GiB RAM alongside 5 other Docker containers. Apache APISIX and Keycloak were deployed on another machine at a different physical location with 4 vCPUs and 16 GiB RAM alongside 14 other Docker containers. To minimise the impact of public network traffic variations, the experiment was repeated 500 times for each of the two approaches and the average response times were calculated. For approach (a), the average response time was 192.36 ms, whereas for (b), it was 228.03 ms. This overhead of 35.67 ms relates to the two additional proxy endpoints introduced in the Data Spine approach as highlighted in Figure 15. We plan to reduce this overhead by deploying all the components of Data Spine at the same local network and by optimising the resource allocation further. Moreover, approach (a) requires getting an access token from every connected platform separately and managing those. Thus, the total response time (time taken for getting the required access tokens plus time taken for calling the services and performing the data model transformation) increases with increase in the number of platforms involved in the composite application. By contrast, in approach (b), only one API call to the EFS is needed to obtain an EFPF access token, reducing the total response time significantly.

## 9. Discussion

The Data Spine is designed and implemented as a federated platform enabler for establishing the EFPF ecosystem, based on the integration, interoperability, and federation requirements, as summarised in Section 3. In this section, we present a summary of architectural considerations, implications and evaluation based on the requirements and from the perspectives of the providers and consumers of smart factory platforms, tools, services, and system integrator users (i.e., usability and multitenancy, platform integration efforts, etc.). This is followed by the discussion on the federated interoperability approach in the Data Spine implementation.

### 9.1. Summary of Architectural Considerations, Implications, and Evaluation

- **Interoperability**: The Data Spine bridges the interoperability gaps between services mainly at three different levels:
  ○ **Protocol Interoperability**: The Data Spine supports two communication patterns:
    1. Synchronous (request-response) pattern
    2. Asynchronous (Pub/Sub) pattern
    While the Data Spine supports standard application layer protocols that are widely used in the industry, it employs an easily extensible mechanism for the inclusion of new protocols. To support lower layer protocols and other IoT networking technologies (e.g., ZigBee, ZWave, BLE, etc.), the Data Spine relies on IoT gateways.
  ○ **Data Model Interoperability**: The Data Spine provides the necessary digital infrastructure and tooling support to transform between the message formats, data structures and data models of different services thereby bridging the interoperability gaps for data transfer.
  ○ **Security Interoperability**: The EFS component of the Data Spine facilitates the federated security and SSO capability in the EFPF ecosystem.
    Furthermore, the mismatch in the interaction approaches followed by different IoT platforms can result in interoperability gaps between them. For example, the services of one platform might expect separate steps for discovery and accessing data, while the services of another platform might provide a querying functionality to retrieve data such that no separate discovery step is needed. Such interoperability gaps between platforms at the levels of “interaction approaches” can be bridged using integration flows for aligning the interactions and the service registry for an additional discovery step, if it is needed.
- **Federation approach**: The EFPF ecosystem architecture follows a federation approach, where the interoperability between different tools/services is established “on-demand” i.e., when required by a use case through an integration flow. As there is no common data model or format imposed, there is no overhead for the system administrators of maintaining such a complex canonical model and on the services to understand it and adhere to it. On the downside, the services are required to create integration flows to interoperate and communicate with each other, in contrast to the interoperability approaches where the services adhere to a common data model defined by the ecosystem and thus, there is no additional overhead of data transformation.
- **Agility and flexibility**: The use of the Data Spine, and the integration flows in particular, to establish interoperability allows the tools/services to be loosely coupled. This allows the tools/services to have neutral APIs not strongly tied to any specific implementation and provides the flexibility to different tools/services to evolve independently. The reliance on APIs as contracts between service providers and service consumers is a standard practice; however, successful collaboration depends upon the former adhering to the semantic versioning [49] standard recommended by the EFPF ecosystem to version their APIs and conveying plans to deprecate/upgrade their APIs to the latter in advance.
- **Usability and multitenancy**: The Data Spine provides an intuitive, drag-and-drop style GUI to the system integrator users to create integration flows with minimal effort. The collaboration of work concerning a particular integration flow among different users is easy to manage as the Data Spine provides a Web-based GUI for creating integration flows. In addition, it provides a multi-tenant authorization capability that enables different groups of users to command, control, and observe different parts of the integration flows, with different levels of authorization.
- **Built-in functionality and tool/service integration effort**: The Data Spine provides connectors for standard communication protocols such as HTTP, MQTT, AMQP, etc., that are widely used in the manufacturing sector. In order to enable transformations among different data models, it provides data transformation processors. For instance, the technology ‘Apache NiFi’ used to realise the IFE provides processors such as JoltTransformJSON, TransformXml, ExecuteScript, ReplaceText, ConvertRecord, etc., for performing data transformation [50]. Thus, the Data Spine takes care of the boilerplate code and facilitates the system integrator users for integrating their services by configuring only the service-specific parts of the integration flows with minimal coding effort.
- **Platform integration effort**: The Data Spine is a cloud-based solution, and therefore, no local deployments are needed to integrate platforms through it. Integration of a platform with the EFPF ecosystem needs alignment of the security roles and access control policies of the platform with those of the ecosystem, registration of its services and creation of integration flows for providing/consuming services.
- **API management**: The system integrator users need to refer to the API specifications of services to create integration flows. The Data Spine provides a service registry component to store and retrieve service metadata including the API specifications. To ensure uniformity across and completeness of the API specifications, the EFPF ecosystem recommends the use of OpenAPI Specification [34] standard for specifying the APIs of services that follow synchronous (request-response) communication pattern and AsyncAPI specification [35] standard for specifying APIs of services that follow asynchronous (Pub/Sub) communication pattern. Thus, this implies that the service providers need to register their services to the service registry and follow the proposed standards to specify the APIs of their services.
- **Modularity and extensibility**: The architecture of the Data Spine has been designed with modularity and extensibility in mind to meet the need for incorporating new tools, services, and platforms in the EFPF ecosystem with minimal effort. The components of the Data Spine are modular in nature and communicate with each other through standard interfaces and protocols. Support for new functionality such as protocols can be added by developing new processors/plugins. In this way, the Data Spine adheres to common industry standards and follows a modular approach to enable the creation of a modular, flexible, and extensible ecosystem.
- **Performance, scalability, and availability**: The EFPF ecosystem makes it easy to integrate new tools/services through the use of Data Spine and promotes reusability. To ensure high performance, high throughput and high availability, the performance critical components of the Data Spine have the capability to operate within a cluster.
- **Maintainability**: The loosely coupled and modular nature of the EFPF ecosystem helps significantly towards its maintainability. A high-quality user documentation of the Data Spine and the smart factory services and tools in the EFPF ecosystem has been published on the ‘EFPF Documentation Portal’ [51].

### 9.2. EFPF Federated Interoperability Approach

From the discussion in the previous section, it is evident that the Data Spine approach aligns closely with the ‘federated approach’ defined by the CEN/ISO 11354 Framework for Enterprise Interoperability [12,13] that is introduced in Section 2. The ‘integrated interoperability approach’ is suitable for a small platform, typically owned and maintained by a single company, in cases where a tight control over the API and the data model is possible. The integrated interoperability approach for small platforms offers better performance, as there is no additional overhead of data transformation. However, this approach does not scale well with the increasing number of tools or services and, therefore, it would not be suitable for an IoT ecosystem consisting of numerous IoT platforms, tools and services provided by different companies.

The ‘unified interoperability approach’ makes use of a common, shared, non-executable metadata model at the ecosystem level and the connected platforms, tools, and services need to adhere to it. The use of a common metadata model makes the process of data transformation easier and less error prone. However, the creation and maintenance of a common metadata model is a very complex task, and its complexity increases with the increasing number of platforms, tools, and services joining the platform ecosystem. In addition, the process of connecting a new platform with the ecosystem becomes more tedious, as it involves mapping of the metadata model of the platform with that of the platform ecosystem. If there are conflicting concepts, the platform’s metadata model might have to be adjusted before it can be integrated with the ecosystem. To tackle the complexity of creating and maintaining such a canonical meta-model, some approaches such as the BIG IoT project [19] make use of community maintained vocabularies such as schema.org as a basis of their metadata models.

The ‘federated interoperability approach’ does not impose the use of a common data model or format and thus, the interoperability needs to be established “on the fly”. To overcome the interoperability gaps at the data level, the parties need to share their ontologies. However, as noted by Schneider, et al. in [18], very few IoT platforms provide formally defined ontologies and due to the lack of familiarity with the Semantic Web technologies, many of the IoT platform owners or providers seem to find it challenging to create such models for their platforms.

The Data Spine approach captures the service/API metadata using standard API specifications. While the Data Spine does not restrict the platform providers to provide documentation of their data models in any specific format, it is agnostic to these formats. The Data Spine can, therefore, be extended to treat the metadata provided in the form of formally defined ontologies differently, so that ontology matching/mapping tools can be used to make the process of data transformation easier. In addition, the EFPF ecosystem can recognize and recommend the use of standard data models specific to the type of data (e.g., OGC (Open Geospatial Consortium) SensorThings [52] for sensor data) or the domain. Moreover, for extending the connectivity options and for making the process of data model transformation easier, new protocol connectors and intuitive data transformation tools can be added to the Data Spine. Finally, the process of creation of composite applications using the tools and services that make use of standards and provide formally defined specifications of APIs and data models can be automated to a higher degree.

## 10. Conclusions and Future Work

Today’s IoT platforms are largely designed as closed, vertically oriented silos with high entry barriers for joining, which hinders overall innovation, market, and economic potentials of digital platforms. Online platforms are seen as stronger drivers in digital society and economy [53] and the creation of innovative, cross-platform solutions and platform ecosystems that offer more added value and transparency is, therefore, necessary. This also requires bridging the interoperability gaps and enabling cross-platform collaboration among existing heterogeneous IoT platforms.

In this paper, we identified the integration, interoperability, and federation requirements for establishing an IoT platform ecosystem and discussed the design and implementation of a federated platform enabler called Data Spine. The Data Spine provides the functionality to federate the identity providers of different platforms, enables SSO functionality at the ecosystem-level and supports interoperability among the services of different platforms, thereby enabling the creation of an extendable ecosystem called EFPF. The Data Spine provides the technological infrastructure for an easy and intuitive creation of cross-platform applications with minimal coding effort. We described the methodology for the integration of synchronous (request-response) as well as asynchronous (Pub/Sub) type of services through the Data Spine from the perspectives of both the service providers and the service consumers. We explained dataflow through the Data Spine using two common use cases from the manufacturing domain. We presented a quantitative evaluation of the Data Spine approach which demonstrated that it offers all the above-listed advantages over the traditional approach where the users must join multiple platforms separately to avail the benefits, at the cost of a reasonable performance overhead, which can be minimized further by fine tuning the deployments and resource allocation. The performance of the Data Spine approach can even be better than the traditional approach in the cases of composite applications involving multiple platforms, as the former requires obtaining only a single access token, whereas the latter requires obtaining as many access tokens as the number of platforms involved in the creation of the composite application.

The current implementation of the EFPF ecosystem is based on four business to business (B2B) platforms focused on supply chains and logistics [5]. The EFPF ecosystem is designed as an extendable solution, ready to integrate new external platforms and their tools/services with the aim to achieve the full implementation stability and market readiness in the future. In the future, we plan to extend the Data Spine to make use of semantic web technologies in order to further automate the process of platform service composition and support automated semantic platform integration.

In parallel to the further implementation of the EFPF ecosystem, the recent European Union (EU) regulations on fairness and transparency in online platform-to-business relationships and proposals for a new Digital Service Act (DSA) [54] and a Data Governance Act (DGA) [55] even further strengthen the data-sharing mechanisms across the EU and shape the business models behind the EFPF ecosystem. Technologically speaking, the anticipated challenges for the EFPF ecosystem refer to the adoption of complementary principles for data sovereignty and trust for data sharing by the International Data Spaces (IDS) initiative. Similar to the EFPF ecosystem, the IDS Reference Architecture Model (IDS-RAM) incorporates the federated identity principles and contributes to the GAIA-X concepts on the future data storage and cloud-related elements [56]. Depending upon the availability and maturity of the GAIA-X Infrastructure Ecosystem, the EFPF ecosystem considers joining forces with both GAIA-X and IDS.

## Figures and Tables

**Table 1:** Summary of the technical profiles of the base platforms.

Technical Aspect	Summary of Adoption by Services
Protocol	Hypertext Transfer Protocol (HTTP)/Representational state transfer (REST), Advanced Message Queuing Protocol (AMQP), Message Queuing Telemetry Transport (MQTT) Minor adoption: WebSockets, Remote Procedure Call (RPC), Common Object Request Broker Architecture (CORBA), RAW
Data Format	JavaScript Object Notation (JSON) Minor adoption: Extensible Markup Language (XML), Open Platform Communications United Architecture (OPC UA) Binary, Proprietary
Data Model	Universal Business Language (UBL), Business Process Model and Notation (BPMN), Open Geospatial Consortium (OGC) SensorThings, OPC UA, Proprietary/Custom Minor adoption: oneM2M, Smart Applications REFerence (SAREF) ontology
Security Method	OAuth 2.0, OpenID Connect, Basic MQTT Authentication Minor adoption: Basic Auth
Identity Provider	Keycloak Minor adoption: Proprietary

**Table 2:** Abstract service description schema of the service registry.

{
“id”: “<unique Id – custom Id or UUID>“,
“type”: “string”,
“meta”: {},
“apis”: [{
“id”: “string”,
“url”: “<base URL of the API>“,
“spec”: {
“mediaType”: “<mediaType type of the API Spec document>“,
“url”: “<URL to API Spec document>“
},
“meta”: {}
}],
“doc”: “<URL to service documentation>“,
“createdAt”: “2020-12-30T15:46:36.793Z”,
“updatedAt”: “2020-12-31T15:46:36.793Z”
}

**Table 3:** Technologies used to realise the conceptual components of the Data Spine.

Conceptual Component	Technology/Software	Version	Source Code
Integration Flow Engine	Apache NiFi [37]	1.11.4	[38]
Application Programming Interface (API) Security Gateway	Apache APISIX [39]	2.3.0	[40]
Service Registry	LinkSmart Service Catalog [41]	3.0.0-beta.1	[42]
Message Broker	RabbitMQ [43]	3.8.5	[44]
EFPF Security Portal (EFS)	Keycloak [33]	3.4.0	[45]
